# Effects of ranolazine on the arrhythmic substrate in hypertrophic cardiomyopathy

**DOI:** 10.3389/fphar.2024.1379236

**Published:** 2024-04-10

**Authors:** James A. Coleman, Ruben Doste, Matteo Beltrami, Alessia Argirò, Raffaele Coppini, Iacopo Olivotto, Betty Raman, Alfonso Bueno-Orovio

**Affiliations:** ^1^ Department of Computer Science, University of Oxford, Oxford, United Kingdom; ^2^ Cardiomyopathy Unit, Careggi University Hospital, Florence, Italy; ^3^ Department of NeuroFarBa, University of Florence, Florence, Italy; ^4^ Meyer Children’s Hospital IRCCS, Florence, Italy; ^5^ Oxford Centre for Clinical Magnetic Resonance Research (OCMR), Radcliffe Department of Medicine, Division of Cardiovascular Medicine, University of Oxford, Oxford, United Kingdom

**Keywords:** hypertrophic cardiomyopathy, ranolazine, drug safety and efficacy, arrhythmic risk, modelling and simulation

## Abstract

**Introduction:** Hypertrophic cardiomyopathy (HCM) is a leading cause of lethal arrhythmias in the young. Although the arrhythmic substrate has been hypothesised to be amenable to late Na^+^ block with ranolazine, the specific mechanisms are not fully understood. Therefore, this study aimed to investigate the substrate mechanisms of safety and antiarrhythmic efficacy of ranolazine in HCM.

**Methods:** Computational models of human tissue and ventricles were used to simulate the electrophysiological behaviour of diseased HCM myocardium for variable degrees of repolarisation impairment, validated against *in vitro* and clinical recordings. S1-S2 pacing protocols were used to quantify arrhythmic risk in scenarios of (i) untreated HCM-remodelled myocardium and (ii) myocardium treated with 3µM, 6µM and 10µM ranolazine, for variable repolarisation heterogeneity sizes and pacing rates. ECGs were derived from biventricular simulations to identify ECG biomarkers linked to antiarrhythmic effects.

**Results:** 10µM ranolazine given to models manifesting ventricular tachycardia (VT) at baseline led to a 40% reduction in number of VT episodes on pooled analysis of >40,000 re-entry inducibility simulations. Antiarrhythmic efficacy and safety were dependent on the degree of repolarisation impairment, with optimal benefit in models with maximum JT_c_ interval <370 ms. Ranolazine increased risk of VT only in models with severe-extreme repolarisation impairment.

**Conclusion:** Ranolazine efficacy and safety may be critically dependent upon the degree of repolarisation impairment in HCM. For moderate repolarisation impairment, reductions in refractoriness heterogeneity by ranolazine may prevent conduction blocks and re-entry. With severe-extreme disease substrates, reductions of the refractory period can increase re-entry sustainability.

## Introduction

Hypertrophic cardiomyopathy (HCM) is the most common genetic heart disease and a leading cause of sudden cardiac death in the young. Pharmacologic strategies to prevent lethal ventricular arrhythmias are limited in HCM, such that implantable cardioverter defibrillators (ICDs) supersede antiarrhythmic drugs as the first-line therapy for sudden cardiac death prevention (Ommen et al., 2020). For every HCM patient that benefits from ICD termination of arrhythmia, there are 7–10 ICDs implanted (Maron et al., 2019), each carrying the risks of surgery and device complications (Lin et al., 2009), such that the development of safe and effective antiarrhythmic drugs for HCM is a priority.

Ranolazine, a late Na^+^ blocking drug used for the control of angina in HCM, has shown efficacy in reducing premature ventricular complex (PVC) burden in symptomatic, non-obstructive HCM patients in the RESTYLE-HCM randomised controlled trial (Olivotto et al., 2018). Real-world analysis has also identified a subgroup of responders to ranolazine for the relief of ventricular arrhythmias, who may be more likely to have paediatric onset HCM (Argirò et al., 2023). This notion is consistent with the finding that young sudden cardiac death victims have less fibrosis at post-mortem examination (Varnava et al., 2000; Gaspari et al., 2021), such that a functional cause of lethal arrhythmias may be present in the early stages of HCM. Functional causes of arrhythmias in HCM include regionally impaired repolarisation (due to enhancement of late Na^+^ and impairment of K^+^ channels) (Sakata et al., 2003; Johnson et al., 2011; Coppini et al., 2013; Lyon et al., 2018) and episodic myocardial ischaemia (Conway et al., 2022; Coleman et al., 2023). Unlike irreversible structural causes of arrhythmias, both of these functional causes may be amenable to ranolazine therapy, which targets repolarisation impairment (Moss et al., 2008; Coppini et al., 2013) and microvascular ischaemia (Argirò et al., 2023).

As potential mechanisms for its antiarrhythmic efficacy, human HCM *in vitro* data has shown that ranolazine, through shortening action potential duration (APD) and favourable effects on Ca^2+^ handling, reduces the incidence of afterdepolarisations (Coppini et al., 2013). Indeed, ranolazine reduced the incidence of spontaneous contractions in a murine model of HCM (Coppini et al., 2017). However, the mechanisms by which ranolazine affects the whole-organ arrhythmic substrate (beyond cellular triggers) are unknown, despite the substrate being critically important for clinically significant arrhythmias such as ventricular tachycardia (VT). *In vivo* investigation of the mechanisms by which ranolazine affects the arrhythmic substrate is largely infeasible in HCM, due to the scarcity of human data and the difficulty of performing invasive mapping protocols in these patients.

The present study therefore used human HCM *in vitro* and ECG data to inform and validate a human multiscale modelling and simulation framework, for the investigation of electrophysiological mechanisms underlying ranolazine effects on the arrhythmic substrate in HCM. Using ectopic pacing protocols in tissue and ventricles with HCM repolarisation impairment, arrhythmic risk was quantified under variable ranolazine concentrations. As APD heterogeneity mediated by late Na^+^ has been shown to form a substrate for VT in animal models (Yamazaki et al., 2022), it was hypothesised that correction of prolonged APDs by ranolazine would reduce refractoriness heterogeneity and the risk of VT. In line with previous *in silico* techniques addressing targeted treatment in inherited cardiomyopathy (Margara et al., 2022), population electrophysiological variability and ECGs derived from biventricular simulations were explored to identify HCM subgroups maximally benefiting from ranolazine.

## Methods

### Modelling cellular electrophysiology

Cardiomyocyte electrophysiology was simulated using the ToR-ORd action potential (AP) model, which is a computational tool that uses biophysically detailed ion channel behaviours to produce APs and Ca^2+^ transients representative of human cardiac electrophysiology (Tomek et al., 2019). The model has been previously validated to reproduce AP behaviours under diseased conditions, including repolarisation impairment in HCM (Doste et al., 2022).

Repolarisation impairment in HCM occurs secondary to ionic remodelling (primarily upregulation of late Na^+^ and downregulation of K^+^ channels), as measured *in vitro* in septal hypertrophy samples from HCM patients undergoing surgical myectomy (Coppini et al., 2013). This experimentally reported ionic remodelling was introduced to the AP model by remodelling ion channel maximal conductances, as in previous works (Passini et al., 2016; Doste et al., 2022). Three patterns of ionic remodelling were considered (Supplementary Table S1): moderate–where only late Na^+^ was upregulated, as reported early in murine HCM (Coppini et al., 2017); severe–where all ionic remodelling components were included, but with K^+^ channel remodelling at half-maximal severity; and extreme–where all ionic remodelling components were included at full severity (Coppini et al., 2013).

Inter-subject variability in cellular electrophysiology was accounted for by repeating all cellular and tissue simulations for 50 different cardiomyocyte AP models randomly sampled from a larger population of experimentally calibrated AP models, as in previous work (Passini et al., 2016). This produced 50 control AP models, paired to 3 × 50 HCM AP models (with moderate, severe and extreme degrees of ionic remodelling).

### Modelling HCM tissue and ventricles

Cardiac electrophysiology was modelled in 2D tissue and 3D biventricular domains to quantify re-entry inducibility and to derive ECGs (see Supplementary Figures S1, S2), using the MonoAlg3D cardiac electrophysiology simulator (Sachetto Oliveira et al., 2018) coupled to the ToR-ORd human ventricular AP model (Tomek et al., 2019). Tissue simulations used a 7.5 × 7.5cm square domain. Human biventricular simulations used a hexahedral mesh derived from an HCM patient with septal hypertrophy, which incorporated transmural heterogeneity (70% endo-, 30% epicardial cells), apex-to-base APD gradients, human conduction velocities and conduction anisotropy, as in previous work (Coleman et al., 2024).

Heterogeneous repolarisation impairment was included in 2D and 3D domains. Despite being experimentally measured in HCM septal hypertrophy samples (Coppini et al., 2013) and confirmed to manifest as prolonged JT_c_ and QT_c_ intervals on the ECG (Johnson et al., 2011), the exact distribution of repolarisation impairment in HCM ventricles is unknown. However, it is thought to be most severe in hypertrophied segments, as this explains some ECG phenotypes (Lyon et al., 2018) and is supported by the association between left ventricular (LV) wall thickness heterogeneity and JT_c_ dispersion (Sakata et al., 2003). Ionic remodelling was therefore included in the domains as a circle/sphere of radius 1.0cm subjected to maximal ionic remodelling, encircled by a border of radius 2.5cm containing a linear gradient in rescaled ionic conductances from non-remodelled up to maximally remodelled tissue. In 2D, this was applied from the centre of the domain, whereas in 3D this was applied from the site of maximal LV endocardial septal hypertrophy. A subset of tissue simulations were repeated for a larger HCM-affected region (radius 1.5 cm encircled by a border of radius 3.0 cm).

Simulations were performed under baseline conditions and under the ionic effects of ranolazine, which had electrophysiological effects on both non-remodelled and HCM-remodelled myocardium.

### Modelling effects of ranolazine

The model of ranolazine was derived from previous computational modelling of the drug (Passini et al., 2017) as informed by *in vitro* IC_50_ measurements (Antzelevitch et al., 2004; Crumb et al., 2016). The IC_50_ for I_Kr_ was adjusted to produce agreement with human *in vitro* experiments at a concentration of 10 µM, where statistically insignificant APD changes of (−30 ± 40) ms were reported in control cardiomyocytes compared to changes of (−120 ± 70) ms in HCM cardiomyocytes paced at 1 Hz (Coppini et al., 2013). Ranolazine was modelled using IC_50_s of 5.9 µM, 77 µM and 30.2 µM for I_NaL_, I_Kr_ and I_Na_, respectively, such that I_NaL_ was predominantly blocked with secondary effects on I_Kr_ and I_Na_.

As experimental models of ranolazine typically administer 10µM as a therapeutic concentration (Coppini et al., 2013; 2017; Yamazaki et al., 2022), yet variable dosage is administered clinically, ranolazine concentrations of 3 µM, 6 µM and 10 µM were considered. These concentrations approximately correspond to dosages of 500 mg bid, 1000mg bid and 1500 mg bid respectively (Jerling, 2006; Tan et al., 2013), where up to 1000 mg bid is used clinically in HCM (Olivotto et al., 2018) and 1500 mg bid has been used in clinical trials (Chaitman et al., 2004).

### S1-S2 pacing protocol

Arrhythmic risk was measured in 2D tissue and 3D biventricular models using an ectopic S1-S2 pacing protocol to attempt arrhythmia induction. Models were initialised with AP model states after 100 cellular S1 beats. Scenarios were then paced for two sinus rhythm beats (S1) at a cycle length of 1000 ms, followed by an ectopic stimulus (S2). A subset of tissue arrhythmic risk simulations were repeated using an S1 cycle length of 700 ms.

In 2D, S1 was simulated as a planar wavefront applied to an edge of the square domain. In the biventricular model, a human physiological activation sequence was used as S1, as in previous work (Cardone-Noott et al., 2016; Lyon et al., 2018). S2 was applied immediately outside of the HCM-remodelled border zone, proximal to the side of S1 in 2D, and in an infero-posterior position in 3D.

Arrhythmia induction was attempted for a range of S1-S2 time intervals, varied in 5 ms increments in the range [APD_CTRL_–T_0_, APD_HCM_ + T_1_], where APD_CTRL_ and APD_HCM_ correspond to APDs in non-remodelled and maximally HCM-remodelled myocardium as measured in cellular simulations. Safety margins T_0_ and T_1_ were set to ensure that (1) ectopic propagation failed at the minimum S1-S2 interval, and (2) conduction was no longer blocked in the HCM-remodelled region at the maximum S1-S2 interval. To facilitate comparisons, T_0_ and T_1_ were identical between simulations with and without ranolazine. To ensure that a minimum of 10 S1-S2 time intervals were always simulated, the maximum S1-S2 interval was increased in a minority of AP models.

On analysis, arrhythmic risk simulation outputs were classified as either (1) the ectopic failed to propagate; (2) the ectopic propagated but no re-entry occurred (PVC); (3) a single re-entry occurred (PVC couplets); or (4) multiple cycles of re-entry occurred (VT). The ranges of S1-S2 intervals at which re-entries were inducible (vulnerable window widths) were used as measures of arrhythmic risk, as measured separately for PVC couplets and VT.

### Clinical ECGs of HCM patients pre- and post-ranolazine

Patients with HCM treated with ranolazine between January 2010 and December 2020 were identified from the Careggi University Hospital Cardiomyopathy Unit (Argirò et al., 2023). Resting 12-lead ECGs recorded before and after ranolazine initiation were available in N = 76 patients at a mean follow-up duration of 17 ± 15 months. Three patients with alternans either pre- or post-ranolazine were excluded, alongside one patient in which the full 12-lead ECG was illegible. A control group consisting of N = 35 HCM patients not initiated on ranolazine with resting 12-lead ECGs on an initial and follow-up visit (25 ± 22 months) were also assembled, selected to exclude those on amiodarone or disopyramide.

Automated ECG delineation software (Martinez et al., 2004) was used to identify points corresponding to the beginning and end of the QRS, and end of the T wave in all 12 leads for each patient. JT intervals were automatically computed and manually reviewed in all 12 leads. Individual leads were excluded in cases of (1) ambiguous T wave end points or (2) excessive noise. JT intervals were rate-corrected according to JT_c_ = JT/RR^1/2^ (Berul et al., 1994). Finally, only leads in the same patient measurable on both the initial and follow-up visit were considered in the analysis.

## Results

### Baseline HCM electrophysiological remodelling phenotypes

**FIGURE 1:**
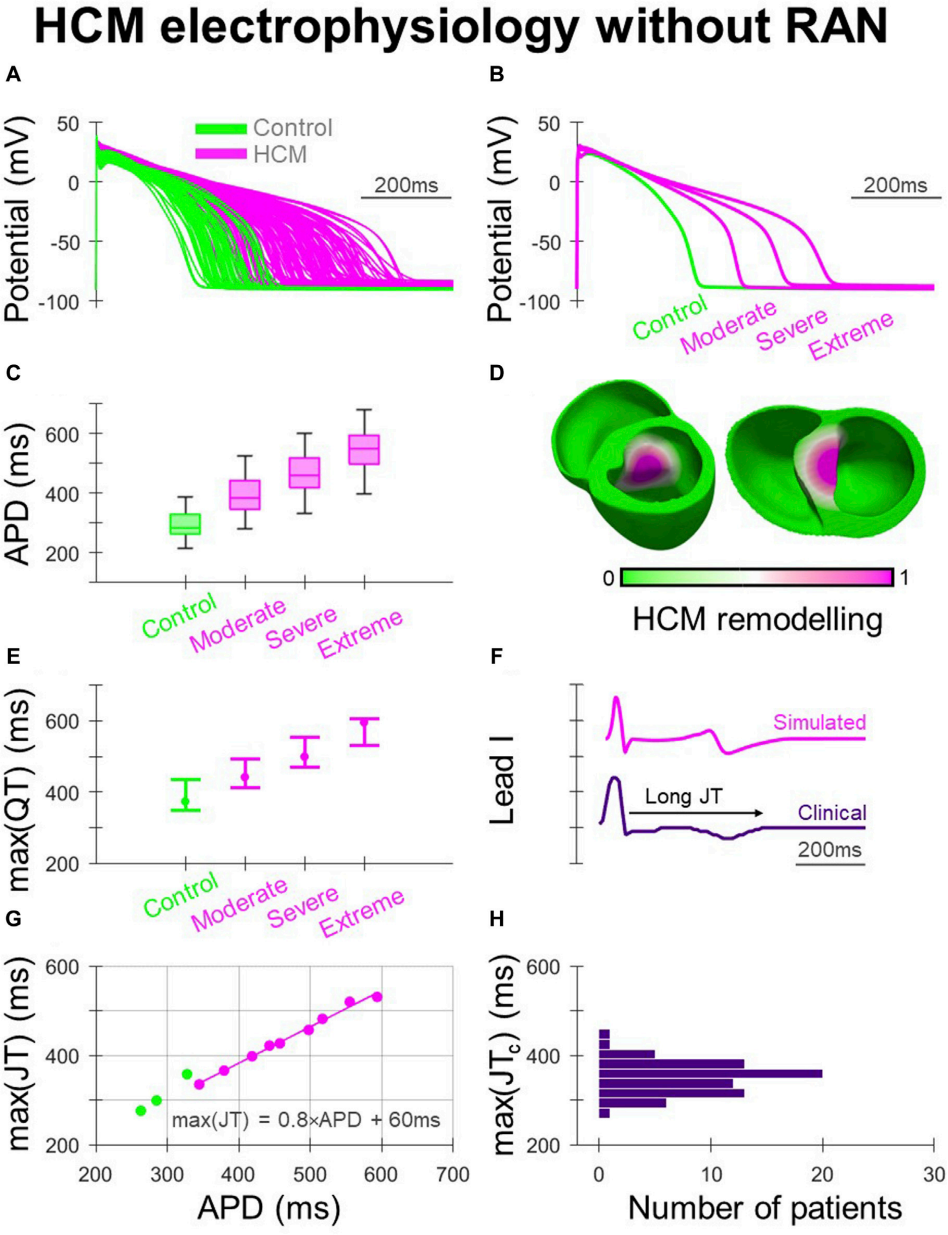
Electrophysiological remodelling in HCM. **(A)** Simulated populations of human ventricular endocardial AP models with non-remodelled electrophysiology (n = 50) paired with HCM-remodelled electrophysiology pooled across moderate, severe, and extreme models (n = 50 each). **(B)** Representative simulated endocardial AP traces. **(C)** APD distributions of control, moderate, severe, and extreme AP models showing increasing APD prolongation. **(D)** Human biventricular model with gradients of HCM electrophysiological remodelling in the region of septal hypertrophy, from which simulated ECGs were derived for the various HCM remodelling severities. **(E)** Maximum QT intervals for the various electrophysiological phenotypes, where AP models corresponding to [25, 50, 75]th APD percentiles were used in biventricular simulations. **(F)** Example lead I ECG traces with prolonged JT intervals, simulated and clinically. **(G)** Maximum JT intervals derived from biventricular simulations, plotted against remodelled cellular APD. **(H)** Maximum JT_c_ intervals measured clinically among HCM patients (N = 72). All simulated results presented at 1 Hz pacing. RAN: ranolazine.

The human ventricular AP models used in simulations are shown in Figure 1A, where HCM electrophysiological remodelling manifested as impaired repolarisation. The variable degrees of HCM remodelling imposed (moderate, severe and extreme) corresponded to the range of APD prolongation demonstrated in Figures 1B, C. When ECGs were derived from biventricular simulations (Figure 1D) with AP models corresponding to the [25, 50, 75]th APD percentiles of their respective HCM electrophysiological remodelling type, increasing degrees of remodelling were associated with increasingly prolonged maximum QT intervals (Figure 1E). This was also reflected in simulated ECG measurements of QT dispersion of 10–40 ms, 40–70 ms, 110–140 ms and 170–200 ms for control, moderate, severe, and extreme HCM remodelling.

Clinically, as both delayed activation and repolarisation contribute to QT prolongation in HCM (Johnson et al., 2011), JT intervals were derived from clinical and simulated ECGs (Figure 1F) to isolate repolarisation effects (Figures 1G, H). Among ECGs derived from biventricular simulations (Figure 1G), the maximum JT interval increased with the APD of the HCM-remodelled region. The relationship between maximum JT and APD was fit according to max (JT) = 0.8×APD +60 ms, to allow relationships between in-tissue arrhythmic risk and APD to be related to the ECG. Cellular APD was only partially translated into regional APDs and JT intervals in the biventricular model due to electrotonic coupling effects. Simulated maximum JT intervals were measured between 280 and 360 ms in controls, and in HCM 340–420 ms, 400–480 ms and 460–530 ms with moderate, severe and extreme remodelling, respectively. Among clinical ECGs (Figure 1H), 64% of patients had maximum JT_c_ > 340 ms (akin to simulations with moderate remodelling), with 6% having maximum JT_c_ > 400 ms (akin to simulations with severe remodelling), similar to JT_c_ measurements made in another HCM study population (Yi et al., 2001).

### Baseline HCM arrhythmic risk

**FIGURE 2:**
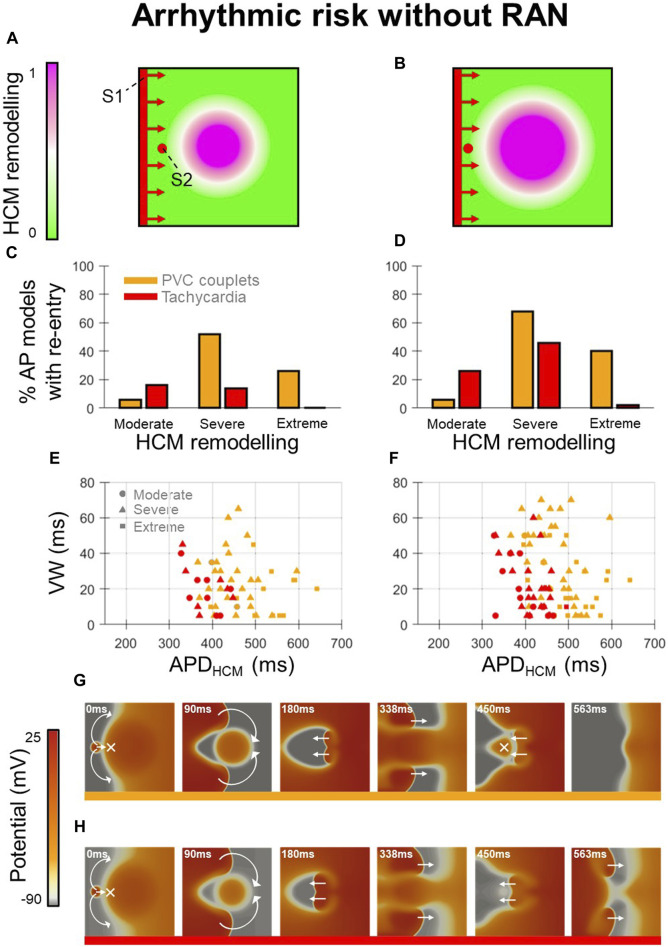
Arrhythmic risk in tissue HCM models without ranolazine. **(A, B)** Tissue domains used to simulate heterogeneous repolarisation for **(A)** medium-sized and **(B)** large regions affected by HCM remodelling. **(C, D)** Percentage of AP models vulnerable to the induction of re-entries (PVC couplets or VT) for moderate, severe, and extreme degrees of HCM remodelling, corresponding to the **(C)** medium-sized and **(D)** large affected regions. **(E, F)** Re-entry vulnerable window width *versus* HCM-remodelled APD for each AP model that had PVC couplets or VT, corresponding to the **(E)** medium-sized and **(F)** large affected regions. Plots are pooled across moderate (circles), severe (triangles) and extreme (squares) degrees of HCM remodelling. Orange symbols denote PVC couplets, while those in red denote VT formation. **(G, H)** Representative tissue simulations after the application of S2, showing the induction of **(G)** PVC couplets, and **(H)** VT (where multiple re-entrant cycles occur). Arrows and crosses denote directions of wavefront propagation and conduction block, respectively. Time elapsed following S2 is denoted in each frame. All results presented at 1 Hz pacing. RAN: ranolazine; VW: vulnerable window width.

Arrhythmic risk at baseline (Figure 2) was quantified for 300 in-tissue scenarios, spread across 150 HCM AP models, and medium-sized and large HCM-remodelled regions (Figures 2A, B). Ectopic S1-S2 protocols applied to these in-tissue scenarios totalled 16,988 simulations of re-entry inducibility including 543 PVC couplets and 216 VT episodes.

Analysis of arrhythmic risk at baseline among the populations of AP models showed that re-entry inducibility was critically dependent on the degree of HCM remodelling (Figures 2C,D). Although re-entry was inducible in a subset of tissue models for all degrees of HCM remodelling, VT was almost only inducible with moderate-severe remodelling (14%–46% of moderate-severe HCM AP models had VT) rather than extreme (0%–2%). PVC couplets were observed most frequently among AP models with severe remodelling (52%–68%), rather than moderate (6%) or extreme (26%–40%). These results suggest that arrhythmic risk initially increases with progression of repolarisation impairment in HCM, then may decline with more advanced ionic remodelling.

Analysis of AP biomarkers pooled across degrees of HCM remodelling yielded remodelled APD as a key determinant of PVC couplet and VT incidence (Figures 2E, F). Compared to the full range of remodelled APDs (280–680 ms), PVC couplets only emerged in a subset (370–640 ms; orange symbols), and VT was inducible only for an even narrower range of APDs (330–490 ms; red symbols). Mechanistically, this is explained by both PVC couplets and VT requiring a threshold extent of APD heterogeneity for unidirectional conduction block of S2 (not satisfied for remodelled APDs <330 ms), but VT imposing a further constraint on remodelled APDs for multiple cycles of re-entry to occur. Secondary to the APD, the HCM-remodelled effective refractory period (ERP) must satisfy ERP< L/v for VT to occur (but not for PVC couplets), where L is the re-entrant circuit length and v is the conduction velocity. This is shown in Figure 2G, where a single re-entry (PVC couplet) occurred due to heterogeneous APDs that enabled conduction block, but further excitation was blocked by refractoriness in the remodelled myocardium ( ERP< L/v not satisfied). Conversely, in Figure 2H shorter remodelled APD still enabled conduction block, but also sustained re-excitation ( ERP< L/v satisfied) such that VT was induced. This condition was responsible for the reduced incidence of VT in cases of extreme vs. severe remodelling, as the long ERP with extreme remodelling precluded multiple cycles of re-entry.

This condition was also responsible for the increased incidence of VT in the large vs. medium-sized HCM-remodelled region (Figures 2C, D), as an increased spatial extent of APD heterogeneity increased circuit length L , such that models with a greater ERP (secondary to larger APD) could now manifest VT. Indeed, VT occurred in models with remodelled APDs of 330–490 ms vs. 330–450 ms in the larger vs. medium-sized HCM-remodelled regions, respectively.

The reduced incidence of PVC couplets in cases of extreme vs. severe remodelling was attributed to the late S1-S2 coupling intervals required to allow the remodelled region to have recovered excitability post-S1. At these late S1-S2 coupling intervals, fast sodium channels were increasingly recovered, such that the conduction velocity of S2 wavefronts approached that of S1, impairing many re-entries. These expected behaviours constituted important validation of the framework, to enable the effects of ranolazine on arrhythmic risk to be subsequently investigated.

### Ranolazine effect on HCM electrophysiology

**FIGURE 3:**
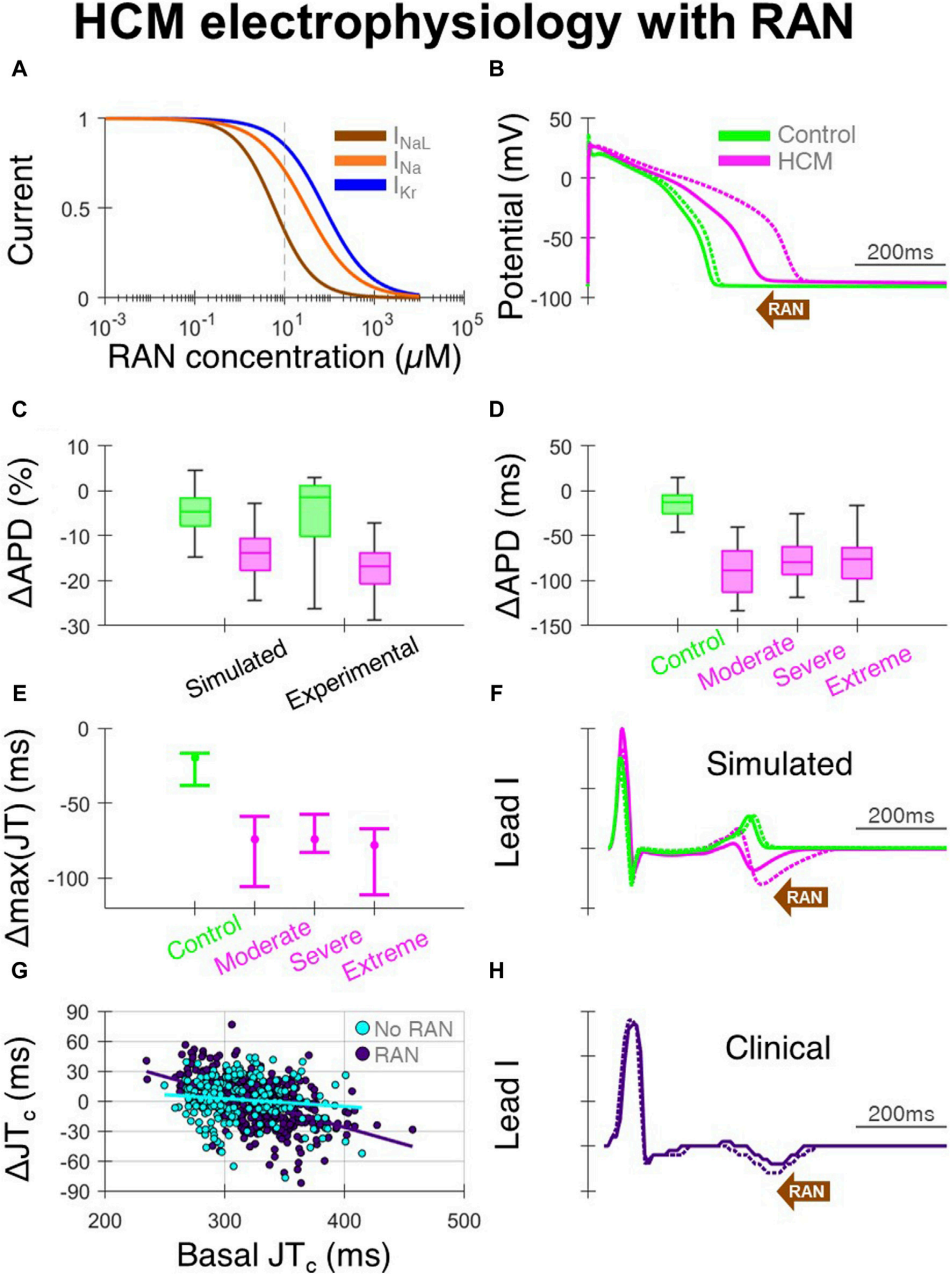
Effects of ranolazine on HCM electrophysiology. **(A)** Dose-response model of ranolazine, where I_NaL_ is primarily blocked with secondary effects on I_Na_ and I_Kr_. **(B)** Representative simulated endocardial AP traces under 10 µM ranolazine (solid) compared with baseline (dashed), showing AP shortening predominantly in HCM. **(C)** Percentage changes in APD under 10µM ranolazine in simulated and experimental setups (Coppini et al., 2013). **(D)** Simulated changes in APD under 10µM ranolazine for control and HCM AP phenotypes, where effects were similar for moderate, severe and extreme degrees of ionic remodelling. **(E)** Changes in maximum JT intervals under 10µM ranolazine for the various electrophysiological phenotypes, where AP models corresponding to [25, 50, 75]th APD percentiles were used in biventricular simulations. **(F)** Representative simulated lead I traces under 10µM ranolazine (solid) compared with baseline (dashed), showing JT shortening predominantly in HCM. **(G)** Change in clinical JT_c_ intervals on follow-up vs. JT_c_ at initial visit, as compared between those initiated with ranolazine (n = 397 leads from N = 68 HCM patients) and those without ranolazine (n = 201 leads from N = 33 HCM patients). **(H)** Example clinical lead I trace after ranolazine (solid) compared with baseline (dashed), showing shortening of the JT interval. All simulated results presented at 1 Hz pacing. RAN: ranolazine.

Ranolazine, due to its predominant block of I_NaL_ (Figure 3A), caused large APD decreases in HCM-remodelled cells, with modest effects in controls (Figure 3B). This APD shortening being confined to HCM cells, without large changes in controls, was validated against that measured in patch-clamp experiments (Coppini et al., 2013) (Figure 3C). Specifically, 10µM ranolazine led to APD changes of −120 ± 70 ms in n = 26 HCM cells from surgical myectomy samples from N = 11 HCM patients (Coppini et al., 2013), compared to −90 ± 30 ms in simulated HCM cells. For controls, APD changes of −30 ± 40 ms occurred in n = 11 cells from N = 4 non-failing non-hypertrophic aortic stenosis patients, compared to −10 ± 10 ms in simulated control cells. Simulated changes in APD were similar across the different HCM remodelling severities (Figure 3D), although slightly larger in cells with moderate remodelling due to the absence of K^+^ channel remodelling. When 10µM ranolazine was applied to biventricular simulations with heterogeneous HCM remodelling (as shown before in Figures 1D, E, where maximum JT intervals were initially prolonged), maximum JT intervals changed by median −70 ms (Figures 3E, F), reflecting correction of impaired repolarisation by ranolazine.

In the clinical ECGs, JT intervals were measurable on the initial and follow-up visit in 397 leads from 68 patients of 864 total possible leads in the ranolazine group, and 201 leads from 33 patients of 420 total possible leads in the no-ranolazine group. Decreases of the JT_c_ interval were observed under ranolazine in leads with longer JT_c_ at baseline (Figure 3G), consistent with acceleration of repolarisation and abbreviation of the T wave (Figure 3H). By comparing changes in JT_c_ intervals in the ranolazine and no-ranolazine groups (Figure 3G), the effects of ranolazine on JT_c_ were estimated. At the lower drug concentration achieved clinically in most patients, JT_c_ intervals changed by −10 to −15 ms in those with 360–380 ms basal JT_c_, and −15 ms to −20 ms in those with 380–400 ms basal JT_c_.

### Ranolazine effect on arrhythmic risk

**FIGURE 4:**
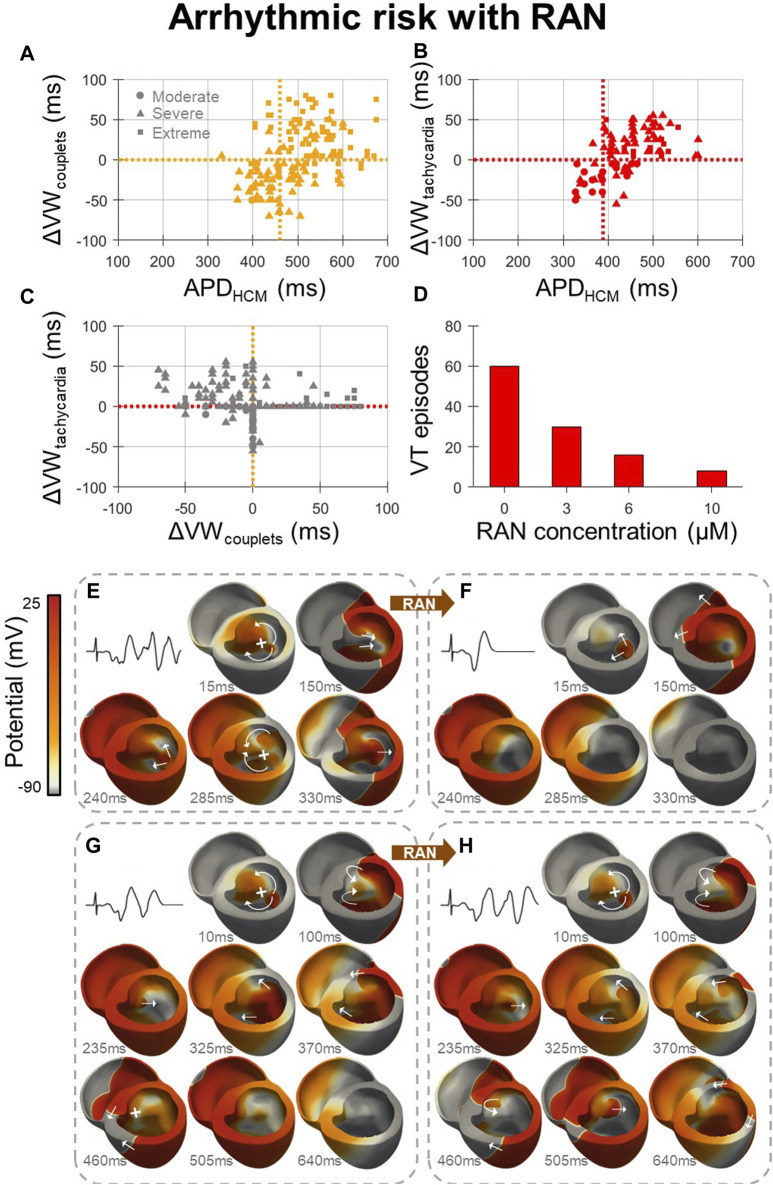
Effects of ranolazine on HCM arrhythmic risk. **(A, B)** The effect of 10µM ranolazine on vulnerable window widths across tissue models, pooled for medium-sized and large HCM-remodelled regions and moderate-extreme degrees of remodelling, for **(A)** PVC couplets and **(B)** VT. Cutoff points in APD for models in which ranolazine should be used (APD < cutoff) for the optimal net reduction in vulnerable window widths are denoted (vertical, dashed). **(C)** Changes in VT vulnerable window widths plotted against changes in PVC couplet vulnerable window widths due to 10 µM ranolazine, showing that many models with a decreased incidence of PVC couplets under ranolazine had a concomitant increased incidence of VT. **(D)** Concentration-dependent effects of ranolazine on the number of VT episodes, when given to models with VT at baseline, as analysed for the medium-sized HCM-remodelled region. **(E)** Representative VT episode inducible at baseline which, **(F)** under 10µM ranolazine, was not inducible due to homogenised repolarisation gradients that precluded conduction block and re-entry. **(G)** Representative PVC couplets inducible at baseline which, **(H)** under 10µM ranolazine, manifested as VT due to the reduced APD and refractory period which enabled further re-entrant cycles to propagate that would otherwise have been blocked at baseline (460 ms frame). Arrows and crosses denote directions of wavefront propagation and conduction block, respectively. Time elapsed following S2 is denoted in each frame. All results presented at 1 Hz pacing. RAN: ranolazine; ΔVW: change undergone in vulnerable window width due to ranolazine.

Arrhythmic risk with ranolazine (Figure 4) was quantified for 600 in-tissue scenarios, spread across 150 HCM AP models, medium-sized and large HCM-remodelled regions, and three drug concentrations (3, 6 and 10 µM). Ectopic S1-S2 protocols applied to these in-tissue scenarios totalled 27,627 simulations of re-entry inducibility, including 1235 PVC couplets and 560 VT episodes.

At all drug concentrations, the effect of ranolazine on arrhythmic risk was critically dependent upon the degree of HCM remodelling, quantified as the HCM APD at baseline, for both the induction of PVC couplets (Figure 4A) and VT (Figure 4B). The inducibility of arrhythmias was decreased by ranolazine in models with a shorter baseline APD, but was increased in models with a longer baseline APD. These effects are related to the dependence of arrhythmic risk on remodelled APD, as presented for HCM models at baseline (Figures 2E, F), where PVC couplets and VT were maximally inducible for an intermediate range of APD prolongation. Ranolazine, which corrected prolonged APDs, pushed some models with moderate-severe remodelling out of this high-risk APD range, but pushed others with severe-extreme remodelling into the high-risk APD range.

Cutoff points in APD were therefore defined, where ranolazine should be used in all models with APD < cutoff, for the optimal net reduction in vulnerable window widths. Computed APD cutoffs were identical across HCM-remodelled region sizes and ranolazine concentrations. Of note, the APD cutoff for PVC couplet prevention (460 ms) was significantly greater than that for VT prevention (390 ms). This is explained by increased VT vulnerable window widths under ranolazine in many models, despite reduced PVC couplet vulnerable window widths (Figure 4C, upper-left quadrant). The suitable APD cutoff for overall antiarrhythmic effect and safety was therefore 390 ms (equivalent to max JT < 370 ms as in Figure 1G). Importantly, however, Figures 4A–C assumed the treatment of ranolazine in all models, independent of baseline arrhythmic risk. Although this was relevant for the safety of ranolazine as an antianginal, for antiarrhythmic purposes it is more relevant to confine the analysis to models with VT at baseline. Models with VT at baseline typically had lower APD anyway (Figures 2E, F), such that 10µM ranolazine applied only to models with VT reduced the total number of VT episodes from 216 to 129 (−40%), and PVC couplets from 543 to 378 (−30%), for analysis pooled over the medium-sized and large HCM-remodelled regions.

Following this strategy of applying ranolazine only to models with VT at baseline, reductions in VT occurred in a dose-dependent manner when analysed for the medium-sized HCM-remodelled region (Figure 4D). Although the maximal reduction in VT was observed with 10 µM ranolazine (−90%), diminishing returns were observed with increasing ranolazine concentrations. Lower concentrations of 3 µM and 6 µM still resulted in comparable VT reductions (−50% and −70% respectively), such that clinical ranolazine dosages could have marked antiarrhythmic effects. For arrhythmic risk in the case of the larger HCM-remodelled region, reductions in VT with this strategy were modest (−20%) even under the maximal concentration of 10 µM ranolazine.

Of note, when 10 µM ranolazine was applied only to models with VT at baseline, *and* APD <390 ms, the net effect on VT was improved for the larger HCM-remodelled region (from −20% to −40%), but slightly worsened for the medium-sized HCM-remodelled region (from −90% to −60%). This was also true at 3µM and 6µM (from −50% and −70% to −40% and −50%, respectively). This reduction in efficacy is explained by AP models which had reduced VT incidence under ranolazine with baseline APD >390 ms, which were not treated when applying the APD <390 ms cutoff.

The mechanism for antiarrhythmic ranolazine effects at low baseline APDs is shown in Figures 4E, F. At baseline (Figure 4E), gradients of repolarisation and refractoriness due to the HCM-remodelled region enabled conduction block following an ectopic stimulus, which then propagated retrogradely and re-entered multiple times (VT episode). When the same model was treated with 10 µM ranolazine (Figure 4F), gradients of repolarisation and refractoriness were homogenised such that the ectopic stimulus propagated throughout the ventricles without conduction block or re-entry (a single PVC).

Conversely, the mechanism for proarrhythmic ranolazine effects at high baseline APDs is presented in Figures 4G, H. At baseline (Figure 4G), a single re-entry occurred but subsequent cycles were blocked by prolonged refractoriness in the HCM-remodelled region (PVC couplets). When the same model was treated with 10 µM ranolazine (Figure 4H), the initial single re-entry occurred almost identically, but later cycles now also conducted as ranolazine decreased refractoriness in the HCM-remodelled region (VT episode).

### Effects of pacing rate

All the previous results considered ventricular pacing at 1 Hz. However, as cutoffs in APD (and the JT interval) were used to optimise ranolazine efficacy, and yet the JT interval is rate-dependent, the effect of pacing rate on the optimal APD/JT cutoff was considered. Arrhythmic risk was therefore further quantified at 1.5 Hz pacing for 300 in-tissue scenarios, spread across 150 HCM AP models for the medium-sized HCM-remodelled region, before and after 10µM ranolazine.

When the effects of increasing pacing rate from 60bpm to 90bpm were analysed, the optimal APD cutoff for VT prevention changed from 390 ms (Figure 4B) to 350 ms, reflecting rate-dependent repolarisation gradients. The rate-dependent decrease in cutoff was compensated for by rate correction of the QT/JT interval, as when the 90bpm vulnerable window analysis was repeated using model-specific APDs corresponding to 60bpm, the 390 ms APD cutoff was preserved.

## Discussion

This study aimed to use human multiscale modelling and simulation to investigate the electrophysiological mechanisms underlying the safety and antiarrhythmic efficacy of ranolazine in HCM, alongside ECG biomarkers associated with beneficial effects. The principal findings of this study are that (i) ranolazine caused decreases of JT intervals in affected leads by accelerating repolarisation; (ii) ranolazine had optimal antiarrhythmic effects in models with moderate repolarisation impairment (maximum JT_c_ < 370 ms); and (iii) ranolazine increased risk of VT only in models with extreme repolarisation impairment.

Although clinical measurements of QT_c_ as measured in a single lead were unchanged by ranolazine on average in HCM (Olivotto et al., 2018), a subset of patients from a larger HCM cohort were reported to have decreased QT_c_ under another late Na^+^ blocking drug, disopyramide (Maurizi et al., 2023). The present study suggests that this is explained by decreases in the JT_c_ intervals of leads with longer baseline JT_c_ under late Na^+^ block. It also suggests that impaired repolarisation and correction by late Na^+^ block may be best observed on analysis of multiple leads, because JT and QT intervals were prolonged non-uniformly among simulated ECG leads, reflecting clinical reports of increased maximum JT_c_ and JT_c_ dispersion in HCM (Yi et al., 2001; Sakata et al., 2003). Indeed, the effects of ranolazine varied among leads in the present study (Supplementary Figure S2), which is consistent with lead-dependent effects on the QT interval identified in previous computational modelling of combined late Na^+^ and K^+^ block with disopyramide in HCM (Coppini et al., 2019). This is explained by repolarisation impairment being heterogeneously distributed in the ventricles secondary to hypertrophy (Sakata et al., 2003; Lyon et al., 2018). The decrease in maximum JT of 70 ms identified in the present study under 10µM ranolazine was consistent with QT_c_ decreases of 56 ms under ranolazine 1000mg bid (Chorin et al., 2016) or 10 ms per 1µM (Moss et al., 2008) reported in congenital LQT3, where the gain-of-function late Na^+^ mutations cause widespread repolarisation impairment. Late Na^+^ block is also reported to reduce QT_c_ in LQT2 patients (Bos et al., 2019), which is relevant as in LQT2 repolarisation impairment is mediated by remodelling in both K^+^ and late Na^+^ channels, like in HCM (Coppini et al., 2013). QT_c_ decreases by late Na^+^ block are lesser in LQT2 than LQT3 (Yang et al., 2021), which is also consistent with the present study in which HCM cells with late Na^+^ and K^+^ remodelling had lesser APD decreases induced by ranolazine than HCM cells with just remodelling in late Na^+^. Finally, simulated changes to the maximum JT interval under ranolazine were driven by both changes in JT_peak_ (−10 ms vs. −30 ms; control vs. HCM) and T_peak_-T_end_ (−10 ms vs. −50 ms) intervals in an AP model-dependent manner, which was attributed to differences in late Na^+^ and K^+^ channel expression between AP models used in the biventricular simulations. Of note, some studies have reported small increases in T_peak_-T_end_ but minimal changes in JT_peak_ intervals in control subjects under ranolazine, reflecting opposing effects of late Na^+^ and K^+^ channel block in early repolarisation (Johannesen et al., 2014).

Antiarrhythmic effects of ranolazine in HCM have been reported clinically, including a reduction in PVC burden in the randomised controlled trial of ranolazine (Olivotto et al., 2018) and the existence of a small real-world subgroup of responders in whom arrhythmic events did not recur after the introduction of ranolazine (Argirò et al., 2023). Moreover, in LQT3, decreases in QT_c_ with late Na^+^ block were associated with reduced arrhythmic events (Mazzanti et al., 2016), such that clinical guidelines recommend late Na^+^ channel blockers (Priori et al., 2013). There is therefore a clinical precedent for the antiarrhythmic effects of ranolazine identified in the present study. However, whereas these simulated antiarrhythmic effects were entirely due to reductions in refractoriness gradients due to late Na^+^ block, ranolazine is also an antianginal, so it is unclear whether clinical antiarrhythmic effects may also be attributed to reduced diastolic compression of the microcirculation and prevention of ischaemia (Coleman et al., 2023). Indeed, in ischaemic non-HCM patients, ranolazine reduced the number and duration of ventricular arrhythmias in the RYPPLE trial (Pelliccia et al., 2015), and ranolazine had antiarrhythmic effects in the first week of treatment in the MERLIN-TIMI trial (Morrow et al., 2007; Scirica et al., 2007). Antiarrhythmic effects like those in the present study are supported by clinical reports in patients without ischaemia (Murdock et al., 2008). This includes the RAID trial, where non-ischaemic patients were enrolled as well as ischaemic patients, and the efficacy in reducing ventricular arrhythmias was not significantly different between these subgroups (Younis et al., 2022). Instead, low-to-moderate risk patients (no atrial fibrillation or other antiarrhythmic drug therapy) derived the most benefit. This might be explained by the presence of structural arrhythmic substrates in higher risk patients not amenable to ranolazine therapy. Indeed, all simulations in the present study considered only a functional rather than structural arrhythmic substrate, whereas fibrotic remodelling is common in HCM, which may explain why ranolazine does not in general prevent VT in HCM (Argirò et al., 2023).

Ranolazine is considered safe for use in HCM (Argirò et al., 2023) and proarrhythmic side effects are not evidenced clinically. Most patients have maximum JT_c_ < 370 ms (72% in our cohort), where the present study predicts only antiarrhythmic effects. Moreover, despite the optimal JT cutoff of 370 ms, ranolazine only had a net proarrhythmic effect for JT cutoffs above 450–490 ms, which far exceeds JT_c_ intervals measured clinically. Clinical studies of ranolazine typically exclude HCM patients with QT_c_ ≥ 500 ms anyway (Olivotto et al., 2018), who are more likely to have prolonged JT_c_ (Johnson et al., 2011). This is because ranolazine is contraindicated in patients with reduced repolarisation reserve at baseline, due to the risk of K^+^ block inducing further QT_c_ prolongation and arrhythmias. Interestingly, the present study suggests that treatment of long QT HCM patients with ranolazine can also be proarrhythmic even when QT_c_ is decreased by late Na^+^ block, due to increases in re-entry sustainability despite reduced repolarisation heterogeneity. Outside of HCM, there is randomised evidence that ranolazine 1000mg bid may be proarrhythmic in some patient subgroups (Younis et al., 2022). As a possible competing mechanism, it is notable that the incidence of early and delayed afterdepolarisations may also depend upon the degree of repolarisation impairment (Coppini et al., 2013), such that proarrhythmic substrate effects of ranolazine at long JT intervals may be counteracted by antiarrhythmic action on cellular triggers.

The present study also motivates reconsideration of whether the QT interval is the optimal index for assessing the risk of drug-induced torsades de pointes in HCM. Both ventricular activation and repolarisation are profoundly delayed in HCM, to a degree that is heterogeneous among patients (Johnson et al., 2011). Although 17% of patients in our cohort had lead II QT_c_ ≥ 480 ms (close to other estimates (Johnson et al., 2011)), 28% had maximum JT_c_ ≥ 370 ms, and the overlap between these groups was suboptimal due to the presence of impaired activation (26% had mean QRS duration ≥150 ms). Some patients with QT_c_ ≥ 480 ms may have a prolonged QRS duration with a normal repolarisation reserve, such that ranolazine could be used safely. Conversely, some patients with QT_c_ < 480 ms could have a normal QRS duration with reduced repolarisation reserve, in whom ranolazine may have torsades de pointes risk. Moreover, as prolonged QRS may be associated with fibrosis in HCM (Park et al., 2020), prolonged QT_c_ in some patients may signify the presence of a structural substrate unamenable to ranolazine. Therefore, the applicability of ranolazine could better be assessed using the JT interval to eliminate the effects of activation delay, despite JT intervals being scarcely reported in HCM.

## Conclusion

The antiarrhythmic efficacy of ranolazine may be critically dependent on the degree of repolarisation impairment, spatial extent of the repolarisation heterogeneity, and ionic remodelling pattern. The partial correction of heterogeneous repolarisation by ranolazine may be antiarrhythmic in most HCM patients (maximum JT_c_ < 370 ms) through the resolution of refractoriness gradients, susceptibility to conduction block and subsequent re-entrant VT. In the setting of extreme repolarisation impairment, ranolazine can be proarrhythmic even when refractoriness gradients are partially ameliorated, through decreasing the maximum refractory period and increasing re-entry sustainability. This highlights the significant complexities involved in the pharmacologic modulation of functional arrhythmic substrates. Both changes to the maximum refractory period, and to refractoriness gradients, are individually important factors in antiarrhythmic drug efficacy and safety, due to the general electrophysiological mechanisms illustrated by Figures 4E–H.

More broadly, the work demonstrates the potential of *in silico* approaches for the investigation of drug safety and efficacy in inherited cardiomyopathies. Multiscale modelling enabled the effects of late Na^+^ block on arrhythmic risk to be explored at a substrate level, extending upon previous *in vitro* and *in silico* experiments in single cells (Palandri et al., 2022), yet avoiding invasive protocols. Such approaches, through identification of ECG biomarkers concomitant with drug safety and efficacy, may advance therapeutic targeting in patients with heterogeneous repolarisation, and strongly motivates better techniques to measure repolarisation heterogeneity *in vivo*. Future work may apply these *in silico* methods to investigate other antiarrhythmic drugs.

## Limitations

The model of ranolazine was calibrated to the *in vitro* effects of ranolazine on human cardiomyocyte APDs (Coppini et al., 2013), where control cells did not experience APD prolongation. However, clinical studies in non-HCM patients report 5–14 ms QT_c_ prolongation under 500–1500mg bid ranolazine (Chaitman et al., 2004; Johannesen et al., 2014). Therefore, the possible effects of APD prolongation by ranolazine was not considered, although these effects are not evidenced in HCM. Ranolazine-induced APD prolongation in non-remodelled myocardium concomitant with APD decreases in HCM-remodelled myocardium may have implications for the arrhythmia mechanisms identified in the present study. Specifically, antiarrhythmic effects secondary to reductions in refractoriness gradients (Figures 4E,F) may be enhanced by a further reduction in refractoriness gradients arising from APD prolongation in non-remodelled myocardium. Conversely, proarrhythmic effects secondary to reductions in the maximum ERP (Figures 4G,H) were determined by ranolazine effects in the HCM-remodelled myocardium, and are unlikely to be affected by APD prolongation in non-remodelled myocardium.

It is further notable that human *in vivo* pharmacokinetic studies have shown that ranolazine is almost entirely metabolised, with three of the metabolites each being 30%–40% as abundant as the parent compound and having variable effects on I_NaL_ and I_Kr_ (Jerling et al., 2005; Chaitman, 2006). Historically, the calibration of computational ranolazine models to *ex vivo* experimental data has led to simulations with unphysiologically large APD/QT_c_ prolongation (Moreno et al., 2013; Passini et al., 2017), due to potent estimates of I_Kr_ block by the parent compound (IC_50_ = 12µM). Much weaker I_Kr_ block by ranolazine metabolites has been reported (IC_50_s > 50µM) (Chaitman, 2006), which is closer to that used in the present study (IC_50_ = 77µM). Further work may consider calibration of ranolazine I_NaL_ and I_Kr_ block using *in vivo* data in both healthy and HCM subjects, to refine representative IC_50_ estimates.

The model of ionic remodelling in HCM cells was assumed to be led by I_NaL_ as supported by murine experiments (Coppini et al., 2017), but the relative contributions of I_NaL_ and I_Kr_ to repolarisation impairment in early human HCM is unknown, despite both contributing to remodelling in surgical myectomy patients (Coppini et al., 2013). Moreover, the spatial distribution of ionic remodelling and its dependence on disease stage is unknown, despite increasing JT_c_ dispersion being associated with increasing LV wall thickness heterogeneity (Sakata et al., 2003). This is relevant to the clinical translatability because the present study showed that the antiarrhythmic effects of ranolazine were dependent on the spatial extent of the repolarisation heterogeneity. Therefore, understanding of the role of repolarisation heterogeneity in arrhythmic risk, and pharmacologic modulation by late Na^+^ block, may be improved by overcoming the significant difficulties in measuring repolarisation heterogeneity *in vivo* (Sahu et al., 2000; van der Waal et al., 2022). Correspondingly, although we identified decreases of the JT_c_ with ranolazine in some leads and this is consistent with the effects of disopyramide in HCM (Maurizi et al., 2023), measures of repolarisation heterogeneity were not addressed due to large numbers of lead exclusions (Sahu et al., 2000). Similarly, the computational study discerned those with heterogeneous repolarisation that may benefit from ranolazine from those with limited benefit, but clinically identifying those with heterogeneous repolarisation remains unsolved.

All biventricular simulations used a single mesh derived from a HCM patient with septal hypertrophy (in accordance with the origin of the biological biopsy samples used in *in vitro* experiments under ranolazine), thus the effect of variable patient-specific geometries on ranolazine effect was not investigated. Conduction velocity in hypertrophied regions was considered as normal as measured invasively in HCM (Van Dam et al., 1972), so possible hypertrophy-associated differences in conduction velocity were not modelled. For simulations with populations of AP models to be computationally feasible, primarily in-tissue simulations were used to investigate arrhythmic risk under ranolazine. Although both antiarrhythmic and proarrhythmic mechanisms of ranolazine observed in tissue were also demonstrated in biventricular simulations, future work may consider the relevance of other ECG biomarkers that are less well translated between tissue and biventricular simulations (e.g., T wave morphology). This study also did not model mechanoelectric feedback or the Purkinje system. As this work focused on investigating the effects of ranolazine on modulating the functional arrhythmic substrate in HCM, other possible structural arrhythmic substrates (diffuse and replacement fibrosis) were not modelled, which may limit ranolazine applicability. Future work may consider modulation of ranolazine effects by concomitant structural remodelling such as fibrosis and disarray. Moreover, although populations of AP models were used, only the ToR-ORd AP model was used as the baseline, as it has been validated for use for modelling repolarisation impairment in HCM. Finally, arrhythmic risk in the present study considered only the transitions from a single applied PVC to PVC couplets or VT; further mechanisms are expected to mediate the initial PVC and the transition from VT to VF, which may also be modulated by ranolazine (Coppini et al., 2013).

## Data Availability

The raw data supporting the conclusion of this article will be made available by the authors, without undue reservation.
