# Supplementary material for: Effects of ranolazine on the arrhythmic substrate in hypertrophic cardiomyopathy
Source: Front Pharmacol. 2024 Apr 10;15:1379236. doi: 10.3389/fphar.2024.1379236 (PMC11039821; doi:10.3389/fphar.2024.1379236)
Supplement: Supplementary file 1 [file DataSheet1.pdf]

Effects of ranolazine on the arrhythmic substrate in hypertrophic cardiomyopathy

James A Coleman[1], Ruben Doste[1], Matteo Beltrami[2], Alessia Argirò[2], Raffaele Coppini[3], Iacopo Olivotto[2,4], Betty Raman[5], Alfonso Bueno-Orovio[1,*]

[1]Department of Computer Science, University of Oxford, Oxford, United Kingdom

[2]Cardiomyopathy Unit, Careggi University Hospital, Florence, Italy

[3]Department of NeuroFarBa, University of Florence, Florence, Italy

[4]Meyer Children's Hospital IRCCS, Florence, Italy

[5]Oxford Centre for Clinical Magnetic Resonance Research (OCMR), Radcliffe Department of Medicine, Division of Cardiovascular Medicine, University of Oxford, Oxford, United Kingdom

*Corresponding author: Alfonso Bueno-Orovio alfonso.bueno@cs.ox.ac.uk, Department of Computer Science, University of Oxford, Oxford, United Kingdom

Supplementary Material

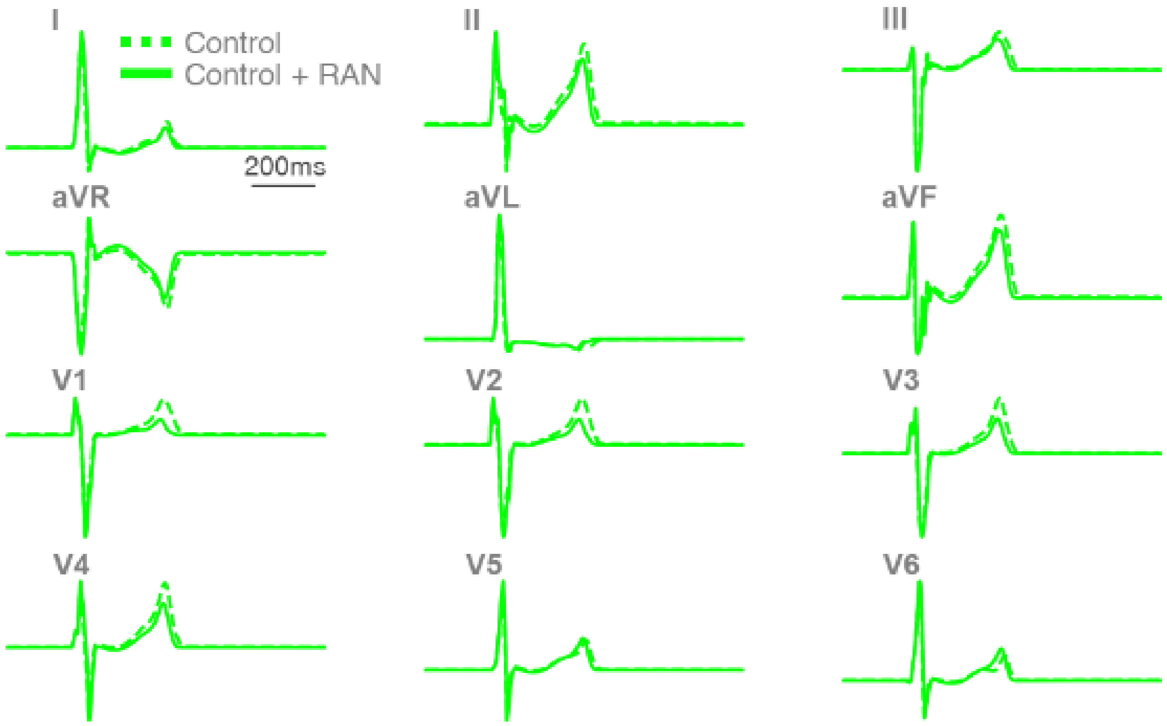

Supplementary Figure S1. Representative simulated ECG without HCM ionic remodelling, before and after 10μM ranolazine. RAN: ranolazine.

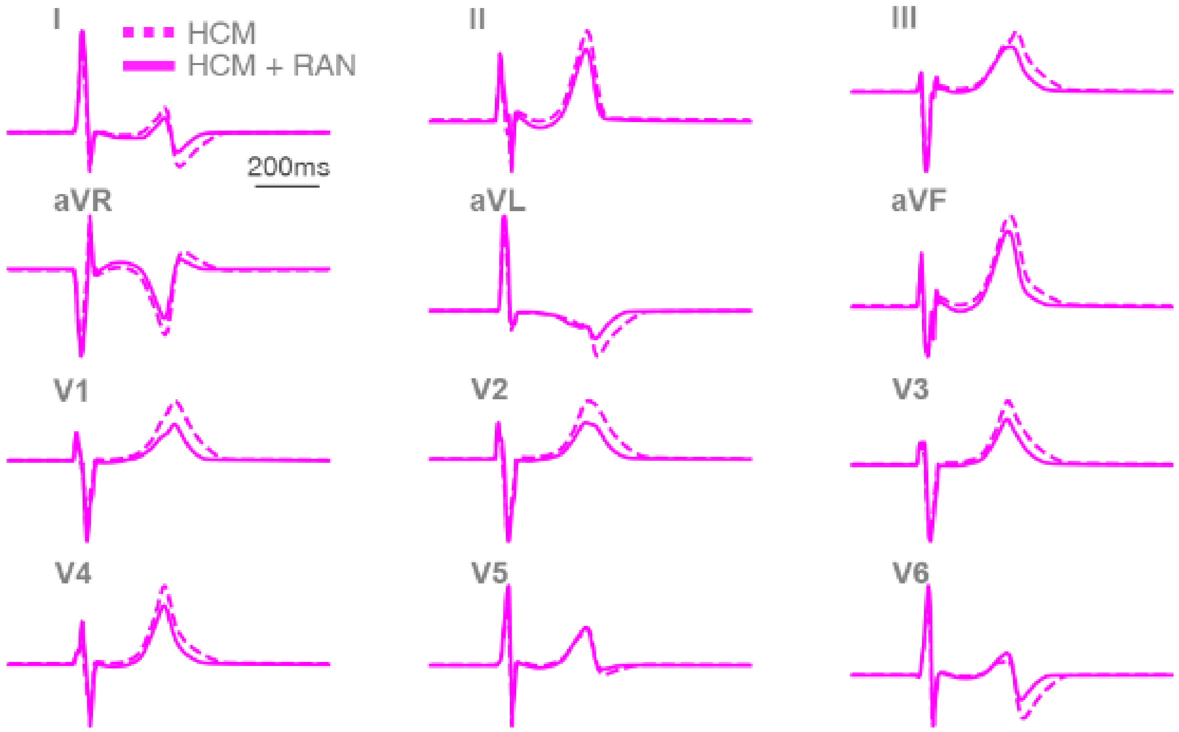

Supplementary Figure S2. Representative simulated ECG with HCM ionic remodelling, before and after 10μM ranolazine. RAN: ranolazine.

Variable/Parameter	Symbol	Change		
		Moderate	Severe	Extreme
Cell volume	V	+0%	+90%	+90%
Troponin-Ca^{2+} affinity	K_{TRPN}	+0%	-50%	-50%
Late Na^+ current	I_{NaL}	+165%	+165%	+165%
Background Na^+ current	I_{Nab}	+0%	+165%	+165%
Inward K^+ rectifier current	I_{K1}	+0%	-15%	-30%
Rapidly delayed K^+ rectifier current	I_{Kr}	+0%	-22.5%	-45%
Slow delayed K^+ rectifier current	I_{Ks}	+0%	-22.5%	-45%
Transient outward K^+ current	I_{to}	+0%	-35%	-70%
Na^+/Ca^{2+} exchange current	I_{NCX}	+0%	+30%	+30%
Na^+/K^+ pump current	I_{NaK}	+0%	-30%	-30%
Ca^{2+} release current	J_{rel}	+0%	-20%	-20%
Ca^{2+} reuptake current	J_{up}	+0%	-35%	-35%
L-type Ca^{2+} current	I_{CaL}	+0%	+10%	+10%
Fast voltage dependent L-type Ca^{2+} time constant	τ_{ff}	+0%	+35%	+35%
Slow voltage dependent L-type Ca^{2+} time constant	τ_{fs}	+0%	+20%	+20%
Fast Ca^{2+} dependent L-type Ca^{2+} time constant	τ_{fcaf}	+0%	+35%	+35%
Slow Ca^{2+} dependent L-type Ca^{2+} time constant	τ_{fcas}	+0%	+20%	+20%
Voltage gating shift in j_{ca}	K_{jca}	+0%	+15	+15

Supplementary Table S1. Model of ionic remodelling in HCM Up/down-regulation of ion currents was achieved through rescaling of maximum conductances, as in previous work (Passini et al., 2016) which was informed by cardiomyocyte currents, protein expression and mRNA data from surgical samples of HCM patients undergoing septal myectomy (Coppini et al., 2013, 2017; Doste et al., 2022).

Supplementary References

Coppini, R., Ferrantini, C., Yao, L., Fan, P., del Lungo, M., Stillitano, F., et al. (2013). Late Sodium Current Inhibition Reverses Electromechanical Dysfunction in Human Hypertrophic Cardiomyopathy. *Circulation* 127, 575–584. doi: 10.1161/CIRCULATIONAHA.112.134932

Coppini, R., Mazzoni, L., Ferrantini, C., Gentile, F., Pioner, J. M., Laurino, A., et al. (2017). Ranolazine Prevents Phenotype Development in a Mouse Model of Hypertrophic Cardiomyopathy. *Circ Heart Fail* 10, e003565. doi: 10.1161/CIRCHEARTFAILURE.116.003565

Doste, R., Coppini, R., and Bueno-Orovio, A. (2022). Remodelling of potassium currents underlies arrhythmic action potential prolongation under beta-adrenergic stimulation in hypertrophic cardiomyopathy. *J Mol Cell Cardiol* 172, 120–131. doi: 10.1016/j.yjmcc.2022.08.361

Passini, E., Mincholé, A., Coppini, R., Cerbai, E., Rodriguez, B., Severi, S., et al. (2016). Mechanisms of pro-arrhythmic abnormalities in ventricular repolarisation and anti-arrhythmic therapies in human hypertrophic cardiomyopathy. *J Mol Cell Cardiol* 96, 72–81. doi: 10.1016/j.yjmcc.2015.09.003
